# Relations between the Diseases of the Nose and the Eye

**Published:** 1890-03

**Authors:** 


### RELATIONS BETWEEN THE DISEASES OF THE NOSE AND THE EYE.

---

Adolph Bronner, M. D., Surgeon to the Bradford, Ky., Eye and Ear Hospital, calls attention, in the *Journal of Laryngology and Rhinology,* to the relations between the diseases of the nose and the eye, in an interesting paper. He says:

"In late years attention has frequently been drawn to the intimate relations between the diseases of the nose and the diseases of the middle ear and throat; but we hear very little of the connection between the diseases of the nose and those of the eye.

"The fact is, that the nose and eye are situated so very near together, that they are in direct connection with one another through the nasal duct; that the venous supply is in direct communication through the frontal veins, the lachrymal plexus, the ethmoidal veins, and others, and that there is a very intimate reflex vasomotor connection. These facts are proof enough that there must be a very intimate relation between the two organs. If we carefully go into the history of many cases of disease of the eye, we do in reality find that they are in close connection with some affection of the nose.

"In most cases of rhinitis we find that the inflammation has spread up the nasal duct, thus causing the mucous membrane of the duct to swell, and preventing the free passage of tears into the nose; or, the inflammation has spread into the lachrymal sac, giving rise to mucocele, and this causes and keeps up inflammation of the conjunctive and cornea. I have, in late years, cured many cases of long standing epiphora, in which there was no stricture and no affection of the lachrymal sac, simply by treating the mucous membrane of the nose.

"Nearly all persons who suffer from chronic hypertrophic rhinitis are also subject to epiphora; the latter varies according to the swelling of the mucous membrane of the nose."

Attention is also called to the fact by other writers, that in most cases of mucocele, or of abscess of the lachrymal sac, we find some affection of the corresponding side of the nares. In most cases of ozæna there is epiphora and conjunctivities, as well as ulcers of the cornea, which are difficult to cure. Careful examination of the lachrymal passages, and removal of any obstruction or inflammation, is recommended before performing any operation on the eye.

After presenting a number of cases, and quoting the remarks of many other observers and writers upon these subjects, he says: "These few facts prove that, in some cases, at least, there is a close connection between the diseases of the nose and of the eye. They prove that, in these days of rapidly growing specialism, we should be careful not to forget that one organ, although it may have its special functions and special diseases, is still a part of the whole, and that we ought always carefully to examine and see if the one disease be not in some connection with some affection of a neighboring organ, or with some constitutional disease.—*The Times and Register.*

